# Look Out

**Published:** 1875-04

**Authors:** 


### LOOK OUT.

And while you are at it, just take a peep at your back yard and be surprised at the nasty mess of garbage that has there accumulated during the winter, now passed. You will find that your not overscrupulous Biddy has found it convenient to deposit her potato-peelings, scraps of meat, bones, stale eggs, kitchen sweepings, and sundry other species of offal that are likely to emanate from every well-regulated family kitchen, upon the surface of the ground, convenient to the door of the culinary department. You will find quite an iceberg there also, produced by the dishwater which is occasionally thrown over the savory mess before described, so that when frozen, it might be firmly attached to the yard, defying the exertions of rats, cats, dogs and other household pets and scavengers from dislodging it.

Now, however, as the first warm rays of an April sun fall upon this variegated heap, you will find little rivulets of putrid water seeking your cellar, cistern, or perchance, your well; in which latter case you will find, too soon, that it will not be *well* for you.

Be it known and remembered, that a well eighteen or twenty feet deep, will act as a drain for the surface of ground, for from twelve to twenty feet all around it. Therefore, whatever of filth may be allowed to accumulate near your well, within this distance, is certain to be represented, to a more or less degree, in the water you drink. Of course, it will appear clear to the eye, and perhaps sweet to the taste, for it has been filtered through the gravel and sand before reaching the well, but it is certain to contain some portion of that poison which produces typhoid fever, and perhaps other equally dangerous diseases.

Then, aside from the danger of this material reaching your well, it is highly deleterious to permit it to be upon the surface of the ground, for there are the unseen seeds of disease and death in the abominable compound. See to it then, that it is speedily and completely removed from your premises, and that the surface of the ground, where it laid, is freely sprinkled with lime.

Be careful, also, to examine your drains. See that they are free and unobstructed, so that no sewer-gas escapes into your apartments—such an occurrence would be very certain to produce typhoid fever in your family. It is a good plan to procure three or four pounds of *sulphate of iron* (copperas) and throw into your water-closets or drains. It should be thrown in whole and undissolved. If repeated several times during the summer, the liability to contagious fever from sewer-gas would be greatly lessened, if not entirely removed.

When it is remembered that all cases of typhoid fever can be traced to a poison induced by sewer-gas, tainted wells, foul cellars and yards, or decomposing vegetable matter, somewhere in the immediate neighborhood, and that it is never known in perfectly cleanly places, provided with wholesome, untainted water, and where perfect sewerage is maintained, the importance of early attending to our instructions are apparent.

Therefore, all hands turn out and thoroughly police cellars, drains, vaults and yards, so that you will be prepared to enjoy a summer as free from sickness as your careful cleanliness can render it.

